# The heterozygous mutation *COL4A4* c.817-1G>A causes Alport syndrome in a Chinese family: a case report

**DOI:** 10.3389/fped.2025.1533638

**Published:** 2025-05-08

**Authors:** Dayan Wang, Xiaobing Li, Kaichao Cheng, Lanjin Zheng, Pengfei Yu, Panjian Lai

**Affiliations:** ^1^ Department of the Pediatric Internal Medicine Ward 3 of the Women’s and Children’s Health Hospital, Jinhua, Zhejiang Province, China; ^2^ Jinhua’s Key Laboratory of Birth Defects Prevention and Treatment, Jinhua, China

**Keywords:** COL4A4, RNA-seq analysis, aberrant splicing, Alport syndrome, case report

## Abstract

**Background:**

Alport syndrome is an inherited glomerular disease that leads to progressive kidney failure, hearing loss, and eye problems. Diagnosis mostly relies on tests of tissue pathology and genetic analysis. This study aims to clarify the role of the *COL4A4* c.817-1G > A mutation in Alport syndrome. The *COL4A4* gene encodes the α4 chain of type IV collagen, which is a key component of the glomerular basement membrane. Mutations in this gene are strongly linked to Alport syndrome.

**Methods:**

We collected clinical data from a 12-year-old boy who had “persistent hematuria for 4 years” and performed a renal biopsy, which was pathologically diagnosed as “Alport syndrome”. We used high-throughput sequencing technology to conduct whole-exome sequencing (WES) and Sanger sequencing for the patient and his parents. Through bioinformatics analysis, we found that the *COL4A4* c.817-1G > A mutation may lead to splicing abnormalities. We extracted RNA from the patients blood and urine samples and used *in vivo* splicing validation to study the impact of the mutation on mRNA.

**Results:**

Our findings show that the COL4A4 c.817-1G > A mutation disrupts mRNA splicing. This mutation affects the splice acceptor site of Intron 13, which is next to Exon 14, causing a 1-bp deletion before Exon 14 and creating a premature stop codon. Consequently, a truncated protein of 273 amino acids is produced, as opposed to the full-length protein of 1,690 amino acids. This finding clarifies the molecular mechanism by which this mutation contributes to Alport syndrome.

**Conclusion:**

Our study finds that the *COL4A4* c.817-1G > A variant may cause autosomal dominant Alport syndrome. Our research expands the mutation spectrum of Alport syndrome while aiding in genetic counseling and diagnosis for affected patients.

## Introduction

Alport syndrome (AS) is a hereditary glomerular disease caused by mutations in the *COL4A3*, *COL4A4*, and *COL4A5* genes. These genes encode the α3, α4, and α5 chains of type IV collagen, respectively (1). This condition is a rare hereditary form of progressive glomerulonephritis, primarily marked by worsening kidney function, conical lenses, and sensorineural hearing loss (1). In recent years, next-generation sequencing technology has significantly improved the detection rates of mutations in *COL4A3*, *COL4A4*, and *COL4A5*. Notably, the carrier frequency of pathogenic mutations in *COL4A3* is as high as 1 in 106, while *COL4A4* has a frequency of 1 in 2,320 (2–4). As a result, the actual prevalence of AS may be significantly higher than the previously reported rates of 1 in 10,000–1 in 5,000 (5). Approximately 85% of patients with Alport syndrome (AS) have X-linked Alport syndrome (XLAS) from mutations in the *COL4A5* gene. The other cases result from mutations in the *COL4A3* or *COL4A4* genes, which cause autosomal recessive Alport syndrome (ARAS) and autosomal dominant Alport syndrome (ADAS) (6). Earlier studies found that Aortic Dissection Symptoms occurred in 1%–5% of Aortic Stenosis (AS) cases, while recent research suggests this number could be as high as 18.9% (2). ADAS has a milder clinical phenotype compared to XLAS (in males) and ARAS. It progresses to end-stage renal disease (ESRD) at a later stage. This study examines the *COL4A4* gene c.817-1G > A mutation and its effect on mRNA splicing. Understanding this process is crucial for uncovering the molecular mechanisms of Alport syndrome. We will extract RNA and perform *in vivo* splicing validation using blood and urine samples from the patient. This research is important because it aims to clarify the abnormal splicing patterns caused by the c.817-1G > A mutation. It will also investigate the potential mechanisms behind these patterns.

## Materials and methods

### Patient and clinical assessments

The proband is a 12-year-old boy who was admitted to Jinhua Maternal and Child Health Hospital because he has experienced persistent hematuria for the past four years. Upon admission, we performed several tests, including a complete blood count, blood biochemistry, a urine routine, 24-hour urinary calcium measurement, bilateral renal ultrasound, renal biopsy, and screenings for vision and hearing. Additionally, we conducted a urine routine and vision and hearing screenings for the proband's parents.

### Trio whole-exome sequencing

Genomic DNA was isolated from peripheral blood samples. Trio whole-exome sequencing (Trio-WES) was conducted at Chigene Genomics in Hangzhou, China. This was performed using a SureSelect Human All Exon kit V6 (Agilent Technologies, Santa Clara, CA) on the Illumina Novaseq6000, achieving an average depth of 100×.The primers used for Sanger sequencing of the *COL4A4* gene c.817-1G > A mutation are as follows:

Primer-F: AAGAGATGATTTCTGAAGGAGATGGA.

Primer-R: CAAGAGATGGCACAATCCTGTCAC.

### Analyze the effects of mutations on splicing through software

Predict the effects of mutations on splicing using the three software tools: SpliceAI, FF, and RDDC.

(1) RDDC: https://rddc.tsinghua-gd.org/
(2) SpliceAI: https://spliceailookup.broadinstitute.org/
(3) FF: https://www.fruitfly.org/seq_tools/splice.html

### Reverse transcriptase PCR analysis

Total RNA was extracted from peripheral blood using the Blood (Liquid Sample) Total RNA Rapid Extraction Kit (RP4001, Bioteke); Total RNA was extracted from urine using the ZR Urine RNA Isolation (R1038, ZYMO RESEARCH) Kit;this was followed by genome digestion and cDNA reverse transcription using the HifairTM 1st Strand cDNA Synthesis SuperMix (11123ES70, YEASEN) kit. Two pairs of nested primers—F1 (5'-CTGGAGAGCCAGGGTTAGTG-3') and R1 (5'-AGTGGAAGAGGTGGAGTCAC-3') as well as F2(5'-TGTGGGAGTAAAGGGGCAAA-3') and R2 (5'-CCAGGATCTCCAACCAGTCC-3')—were designed to amplify the exon 13–16 of COL4A4. The system was heated to 95°C for 5 min, followed by PCR amplification using PrimerSTAR MAX DNA Polymerase (R045A, TaKaRa) at 95°C for 30 s, 57°C for 30 s, and 72°C for 90 s (35 cycles), and then 72°C for 5 min. Finally, the second-round PCR products were sequenced by Sanger sequencing to verify the results. The wild-type product obtained by PCR amplification was directly subjected to Sanger sequencing, and the mutant product was TA cloned using Hieff Clone® Zero TOPO-TA Simple Cloning Kit (10908ES20, YEASEN), and then a single clone was selected for Sanger sequencing.

## Results

### Case report

**Figure 1:**
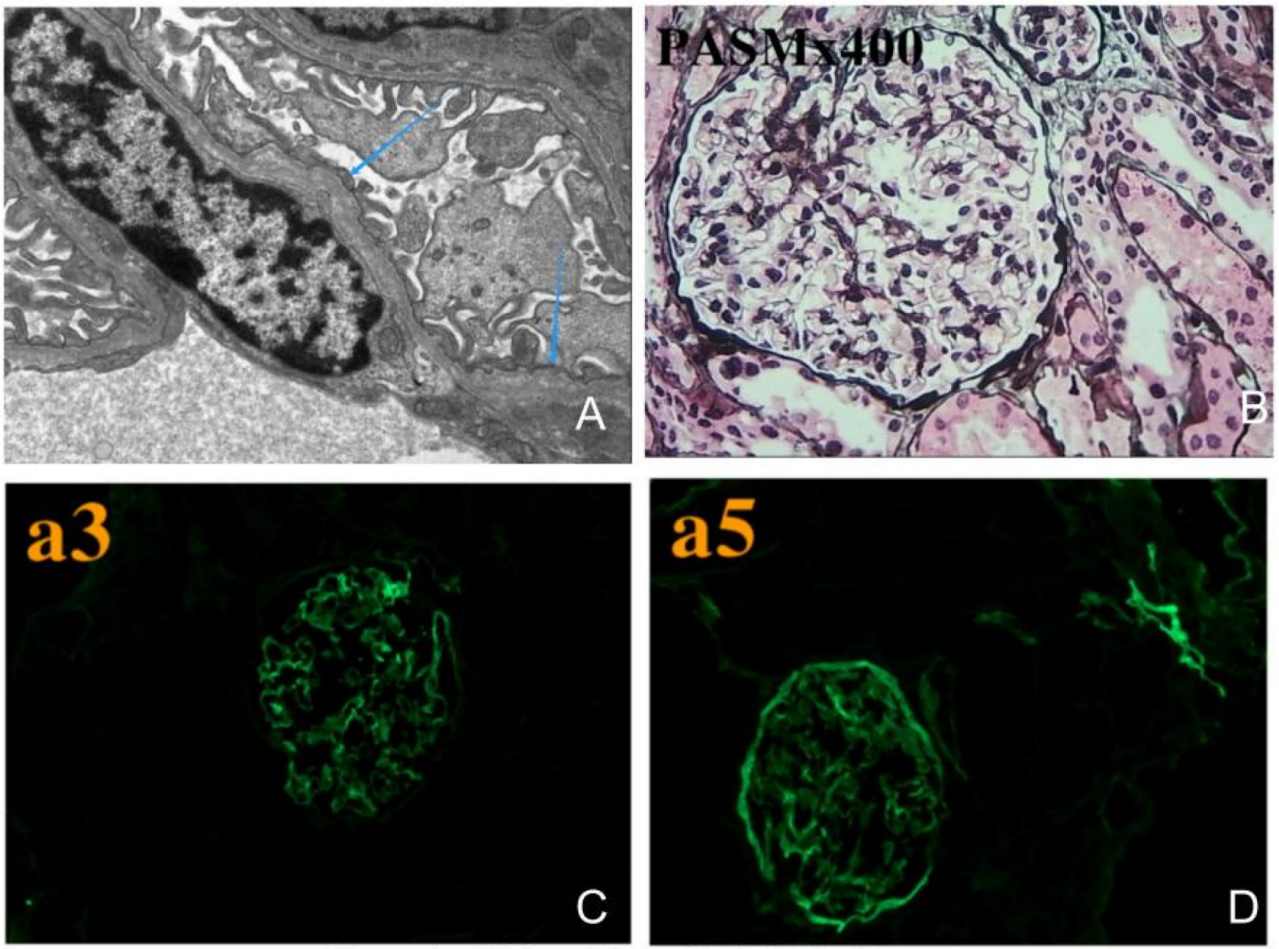
Kidney biopsy pathological images. **(A)** Electron microscopy shows that the basement membrane varies in thickness, with certain areas of the dense layer thickening and irregular patterns that look like fractures and a spider web. **(B)** Light microscopy after PASM staining shows uneven coloration of the basement membrane. **(C)** Immunofluorescence testing shows segmental loss of α3 in both the glomerular and renal tubular basement membranes, **(D)** while α5 is also lost in the glomerular basement membrane, Bowman's capsule, and renal tubular basement membrane.

The subject of this case is a 12-year-old boy whose parents are not related by blood. The patient's mother has a history of hematuria; however, her renal function, hearing, and vision are all normal. The patient is the first child of his mother, born at full term via a normal delivery, weighing 3,400 grams, and his growth and development are in line with those of his peers. When he was 8 years old, the patient was diagnosed with ongoing dark brown blood in his urine. Repeated urinalysis revealed hematuria, with a positive three-plus result for blood and urinary red blood cell counts ranging from 1,000 to 5,000/µl. Renal function tests and kidney ultrasound showed no abnormalities. No hearing impairments or ocular abnormalities were noted. The patient underwent a kidney biopsy at the age of 8. The renal pathological examination under electron microscopy revealed variable thickness of the basement membrane, ranging from about 100–450 nm (Figure 1A). It also exhibited segmental thickening of the dense layer and irregularities that resembled tears and a spider web.Immunofluorescence testing revealed a regional loss of α3 in both the glomerular and renal tubular basement membranes. Additionally, there was a regional loss of α5 in the glomerular basement membrane, Bowman's capsule, and renal tubular basement membrane (Figures 1C,D). Light microscopy after PASM staining demonstrated uneven staining of the basement membrane (Figure 1B). We have followed up with the patient for four years. The child primarily exhibits hematuria, with no proteinuria or kidney function damage. Therefore, no further special treatment has been given.

### Identification of a heterozygous splicing site variant in *COL4A4*

**Figure 2:**
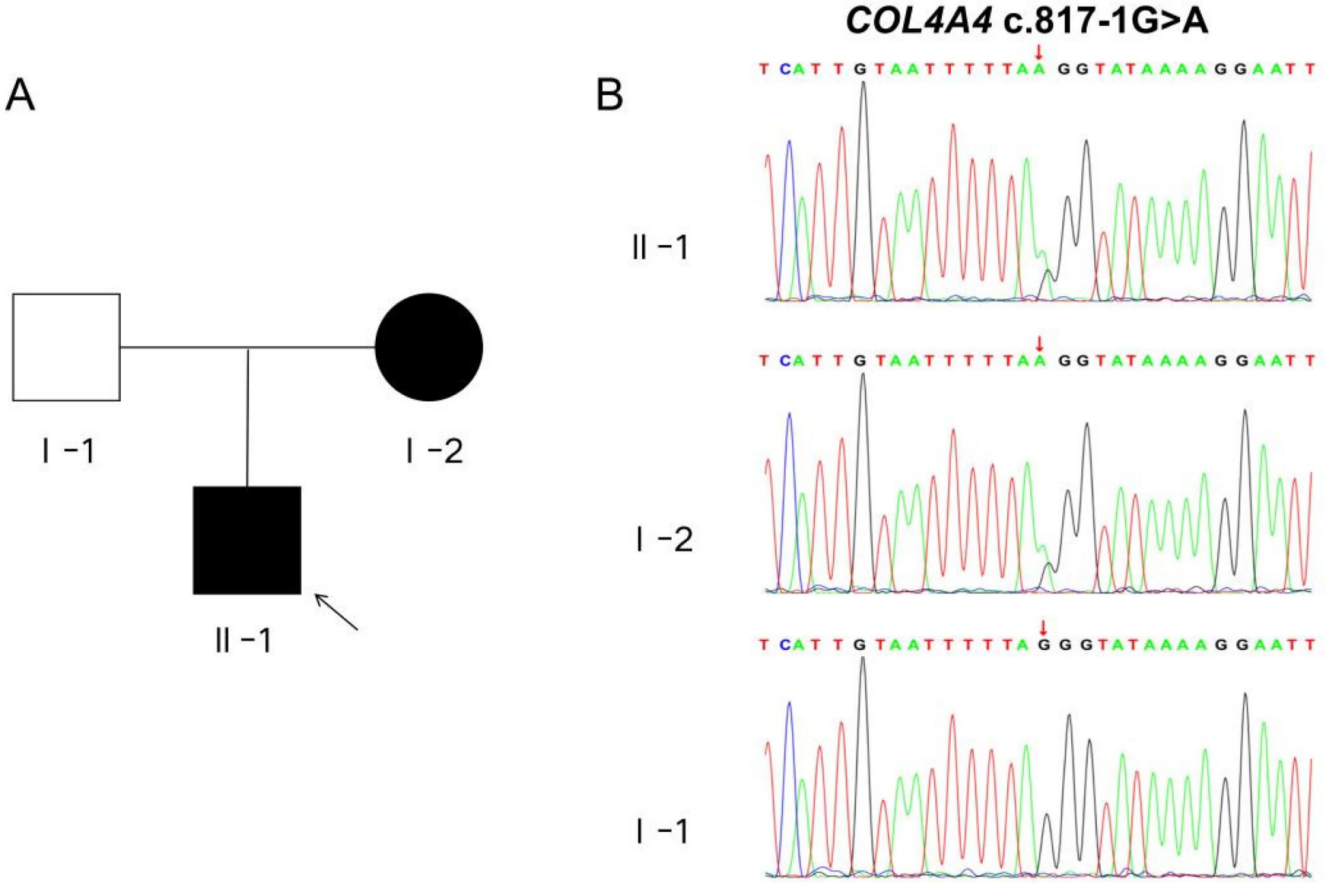
Pedigree chart and sanger sequencing validation diagram. **(A)** The schematic representation of the patient's familial pedigree shows that I-1 represents the father, I-2 represents the mother, and II-1 represents the proband. The white shading indicates an unaffected family member, whereas the black shading indicates an affected member. **(B)** The Sanger sequencing chromatogram of the *COL4A4* gene for the family is presented below. The variant c.817-1G > A in *COL4A4* was identified in both II-1 and I-2.

Because the kidney biopsy revealed “Alport syndrome” and the child's mother has hematuria, we conducted whole-exome sequencing on the child and his parents to investigate the genetic factors responsible for this disease. In the genetic testing, two mutations associated with the patient's phenotype were identified: *COL4A3* c. -81 (exon 1) G > C and *COL4A4* c. 817-1 G > A mutations. Both mutations were found to be present in public databases, including the Exome Aggregation Consortium (ExAC), the 1,000 Genomes Project (MAF), and the Genome Aggregation Database (gnomAD).The *COL4A3* c.-81 G > C mutation was present only in the patient and his healthy father, suggesting it is unlikely to be responsible for the disease. Conversely, the *COL4A4* c.817-1G > A mutation was found in both the child and the mother, providing evidence for the co-segregation of the variant with the disease. Furthermore, the three software tools (SpliceAI, FF, RDDC) show that the *COL4A4* c.817-1 G > A mutation affects splicing, while the *COL4A3* c.-81 G > C mutation is unlikely to do so. Thus, the *COL4A4* c.817-1 G > A mutation is likely the main pathogenic gene causing the child's condition. We performed Sanger validation of the *COL4A4* c.817-1 G > A mutation using blood samples from the proband and his parents. To achieve this, we designed specific primers flanking the target region and amplified the target fragments using PCR.Our findings indicate that the proband and his mother carry the heterozygous *COL4A4* c.817-1 G > A mutation, while the father carries two copies of the wild-type allele (Figure 2).

### Effect of the *COL4A4* c.817-1 G>A variant on splicing

**Figure 3:**
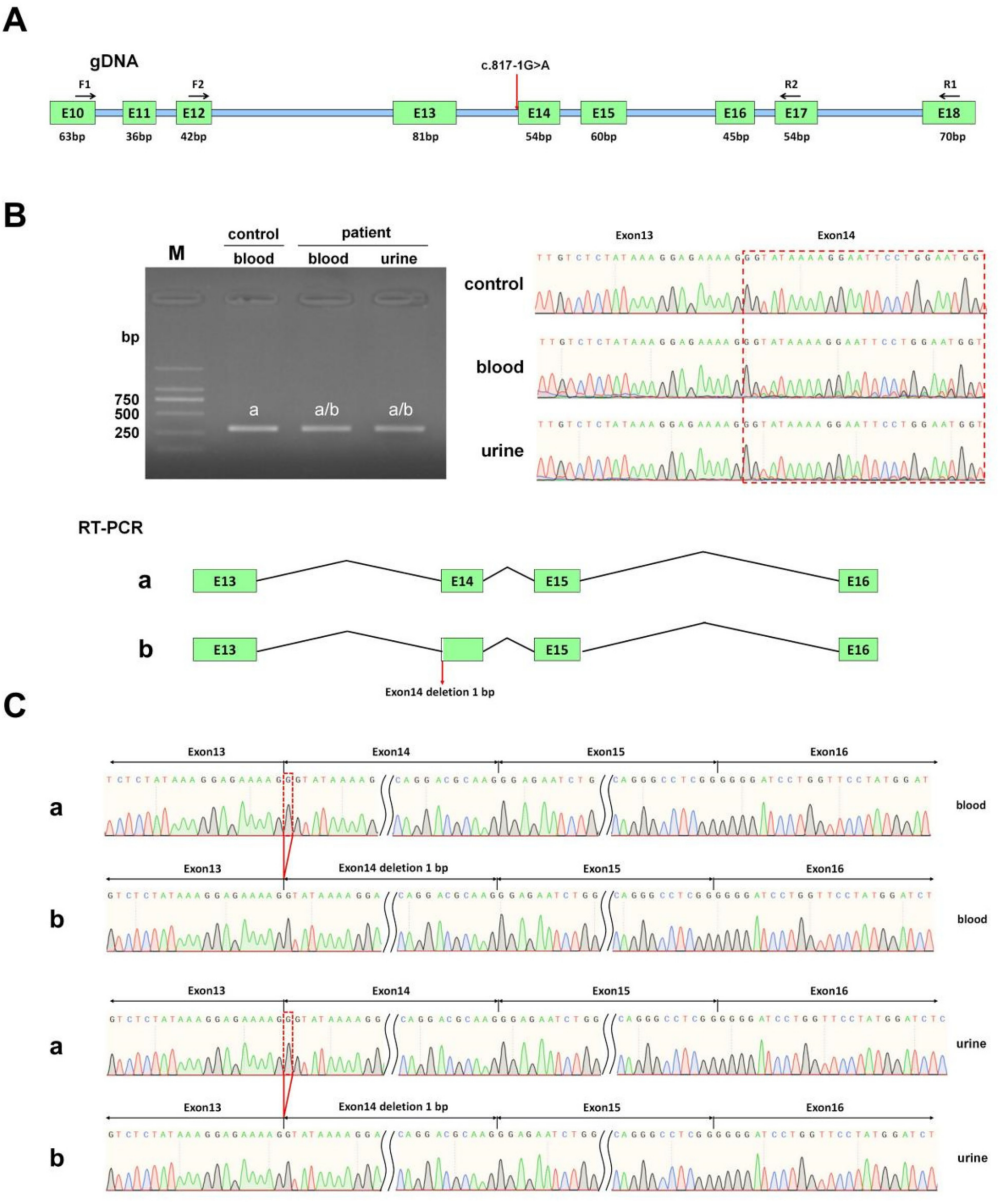
*In vivo* splicing analysis of the impact of the COL4A4 c.817-1G > A mutation on mRNA splicing. **(A)** This section includes a diagram that illustrates primer design and splicing, where the red arrow indicates the mutation site. **(B)** The section presents the RT-PCR product agarose gel electrophoresis image, preliminary sequencing results, and a splicing diagram. In this diagram, the band from the normal sample is labeled as “band a,” while the bands from the patient's blood and urine samples are labeled as “band a” and “band b,” respectively. **(C)** This part presents the sequencing results corresponding to splicing bands “band a” and “band b” obtained from the blood and urine samples.

To confirm the pathogenicity of the *COL4A4* gene mutation c.817-1 G > A, we performed *in vivo* splicing experiments using blood and urine samples from the patient, along with blood samples from healthy volunteers as a control group (Figure 3A). After PCR amplification, electrophoresis revealed a single band in all three samples, which were subsequently purified before being subjected to Sanger sequencing. The sequencing results indicated that normal individuals had only one splicing variant. This variant matched the expected size of 300 bp and is referred to as band a (Figure 3B). The sequencing results from the patient's blood and urine samples showed overlapping peaks, contrasting with the distinct peaks observed in normal samples (Figure 3B). Therefore, we purified the PCR products, performed TA cloning, and conducted Sanger sequencing. The results identified two splicing variants, referred to as band a and band b, as illustrated in Figure 3B. The sequencing results show that band a, found in healthy individuals, corresponds to normal splicing. The patient's blood and urine samples revealed band a, which is a normal splicing variant, and band b, which is an abnormal splicing variant. Sanger sequencing showed that this abnormal splicing variant has a one-base pair deletion on the left side of exon 14 (Figure 3C).

*in vivo* studies showed that the mutation c.817-1G > A disrupts normal mRNA splicing by affecting the splice acceptor site of Intron 13, which is adjacent to Exon 14, leading to a 1 bp deletion in Exon 14. This deletion is reflected in the cDNA and protein sequences as c.817delG p.Gly273Val fs*2. The mutation changes the reading frame and introduces a premature termination codon (PTC) in Exon 14. This alteration may result in a truncated protein of about 273 amino acids, whereas the protein encoded by COL4A4 is expected to be approximately 1,690 amino acids long.

### Interpretation of the variants based on the ACMG guidelines

The *COL4A4* c.817-1 G > A variant is a new splicing variant, and its harmful effect on splicing has been demonstrated through *in vivo* functional experiments. Based on the guidelines from the American College of Medical Genetics and Genomics (ACMG), the *COL4A4* c.817-1 G > A variant is classified as pathogenic.The following criteria establish the pathogenicity level, which is calculated as PM2 + PP1 + PS3 moderate = P.

PM2: The mutation c.817-1G > A is not found in the normal controls of the gnomAD database.

PP1: The mutation co-segregates with the disease in the family, where the mutation has been detected in two affected individuals.

PS3: *in vivo* functional validation indicates that the *COL4A4* c.817-1 G > A variant leads to a 1 bp deletion at the beginning of Exon 14. This deletion alters the reading frame, producing a premature termination codon (PTC) within Exon 14, which may result in a truncated protein consisting of 273 amino acids.

## Discussion

ADAS was first reported by Jefferson et al. in 1997 (7), proposing that mutations in the *COL4A3* and *COL4A4* genes can lead to a spectrum of glomerular basement membrane diseases, including atypical Alport syndrome (ADAS) and familial benign hematuria. In 2004, Pescucci et al. (8) examined four families with autosomal dominant Alport syndrome (ADAS) and confirmed that mutations in the *COL4A4* or *COL4A3* gene cause the condition. They discovered that 22 patients displayed a range of phenotypes. These varied from non-progressive isolated microscopic hematuria to end-stage renal disease and advanced renal failure by the age of 50. Studies show that 12.5%–33.3% of ADAS patients may progress to end-stage renal disease (ESRD), and the incidence of ocular and auditory problems in ADAS patients is relatively low. Current research reveals that between 50.0% and 65.2% of patients have proteinuria, 12.0%–24.3% progress to ESRD, and 2.4% to 4.0% develop ESRD before age 41, with a median renal survival time of 70 years. Furthermore, 3.0%–8.4% of patients experience sensorineural hearing loss, and approximately 2.7% suffer from ocular damage (9–11). Over the past decade, the introduction of next-generation sequencing (NGS) has greatly accelerated the process of genetic diagnosis for Alport syndrome (AS) (12). New sequencing technologies may reveal that *COL4A4* mutations are more common in the population than previously thought. However, physicians have recognized the limitations and challenges of NGS. This is especially true for patients who have a clinical diagnosis or strong suspicion of AS. While some patients have completely negative NGS results, others have uncertain significance variants (VUS) in the *COL4A3* to *COL4A5* genes, which complicate genetic diagnostics and the analysis of genotype-phenotype correlations. These ambiguous variants create difficulties for genetic diagnostics and genotype-phenotype correlation analyses. Research shows that abnormal splicing, which accounts for about 13%–25% of harmful variants in the *COL4A3* to *COL4A5* genes, can occur due to both typical and atypical mutations at splicing sites, including deep intronic changes and exon substitutions (13, 14). Consequently, evaluating how VUS influences *COL4A3* and *COL4A5* transcripts is vital for uncovering new potential splicing variants.

In our study, utilizing whole exome sequencing (WES) and Sanger sequencing, we identified a novel mutation in *COL4A4* (c.817-1 G > A) in an affected individual from a Chinese family with a history of Alport syndrome (AS). According to ACMG guidelines, this mutation is classified as a Variant of Uncertain Significance (VUS). It is located at a splicing site in intron 13, which falls within the “Class I mutation region” that affects splicing. The splicing impact of this mutation has been predicted to be positive, with a probability of up to 90%, according to bioinformatics databases such as SpliceAI, FF, and RDDC. Analysis of the *COL4A4* gene mRNA from the patient's blood and urine showed a mutation. This mutation alters the splice acceptor site of Intron 13. It is located next to Exon 14 and involves a 1 bp deletion on the 5’ side of Exon 14. This mutation is represented at the cDNA and protein levels as c.817delG p.Gly273Val fs*2. This variant changes the reading frame, resulting in a truncated protein that is 273 amino acids long, whereas the normal gene produces a protein of 1,690 amino acids. The proband's clinical phenotype displayed persistent microscopic hematuria and visible hematuria. Kidney pathology revealed typical segmental thickening of the basement membrane, segmental tearing, and mesh-like changes on electron microscopy. Immunofluorescence indicated localized loss of the α3 chain in the glomerular and tubular basement membranes, while the α5 chain showed loss in the glomerular basement membrane, Bowman's capsule, and tubular basement membrane. As a result, the diagnosis of Autosomal Dominant Alport Syndrome (ADAS) was made for the patient, attributed to the *COL4A4* (c.817-1 G > A) mutation. The patient's mother has the same genetic mutation, but her hematuria is mild and does not present with gross hematuria. Additionally, her symptoms are less severe than those of the patient. These differences might arise from variations in gender, hormonal levels, environmental influences, and genetics.

Wang, X. and colleagues analyzed mRNA in urine and skin fibroblasts. They successfully identified intronic splicing variants responsible for Alport syndrome, addressing the problem of negative results from exon sequencing. Based on their findings, they recommend urine mRNA analysis as the most effective non-invasive diagnostic method (15). The research by Yanqin Zhang et al. (16) found that analyzing mRNA from urine assists in identifying abnormal splicing associated with unclassified variants in Alport syndrome genes. They discovered nine unclassified variants that change mRNA splicing in the *COL4A3* to *COL4A5* genes by analyzing mRNA from blood and urine. We conducted an analysis of mRNA from both blood and urine samples of the male patient, extracting RNA and performing *in vivo* RNA splicing verification. We amplified the PCR products, then performed TA-cloning and selected a single clone for Sanger sequencing. The sequencing results of the urine sample were consistent with those of the blood sample, both showing a 1-bp deletion on the left side of Exon 14. Therefore, urine mRNA analysis and mRNA analysis from blood samples have identical accuracy. Blood and urine mRNA analysis can be routinely used for the genetic diagnosis of AS, allowing researchers to classify variants from “unclassified” to “pathogenic” or “benign,” thus enhancing diagnostic accuracy. Urine samples are analyzed for mRNA, which is a non-invasive and easily accessible method well-suited for diagnosing genetic diseases.

Alport syndrome is a chronic kidney disease. Detecting and diagnosing it early can greatly increase the chances of preventing its onset and progression in patients (17). Early detection and diagnosis can effectively increase the chances of preventing offspring from developing AS and its progression. In summary, we identified a novel heterozygous mutation in *COL4A4* (c.817-1 G > A) in an autosomal dominant AS family through whole exome sequencing (WES), Sanger sequencing, and *in vivo* splicing verification. This study examines a new mutation that helps explain the causes of AS, increases the variety of known mutations related to the condition, and assists in genetic diagnosis and counseling for patients with kidney diseases.

## Data Availability

The datasets presented in this study can be found in online repositories. The names of the repository/repositories and accession number(s) can be found in the article/Supplementary Material.
